# Muscle strength and prediabetes progression and regression in middle‐aged and older adults: a prospective cohort study

**DOI:** 10.1002/jcsm.12905

**Published:** 2022-01-23

**Authors:** Shanhu Qiu, Xue Cai, Yang Yuan, Bo Xie, Zilin Sun, Duolao Wang, Tongzhi Wu

**Affiliations:** ^1^ Department of General Practice, Zhongda Hospital, School of Medicine Southeast University Nanjing China; ^2^ Institute of Diabetes, School of Medicine Southeast University Nanjing China; ^3^ Department of Endocrinology Shenzhen People's Hospital (The Second Clinical Medical College of Jinan University) Shenzhen China; ^4^ Department of Nursing Management, Zhongda Hospital, School of Medicine Southeast University Nanjing China; ^5^ Department of Endocrinology, Zhongda Hospital, School of Medicine Southeast University Nanjing China; ^6^ Department of Clinical Sciences Liverpool School of Tropical Medicine Liverpool UK; ^7^ Adelaide Medical School and Centre of Research Excellence (CRE) in Translating Nutritional Science to Good Health The University of Adelaide Adelaide SA Australia

**Keywords:** Muscle strength, Prediabetes progression, Prediabetes regression, Cohort

## Abstract

**Background:**

Prediabetes progression is associated with increased mortality while its regression decreases it. It is unclear whether muscle strength is related to prediabetes progression or regression. This study investigated the associations of muscle strength, assessed by grip strength and chair‐rising time, with prediabetes progression and regression based on the China Health and Retirement Longitudinal Study (CHARLS) enrolling middle‐aged and older adults.

**Methods:**

We included 2623 participants with prediabetes from CHARLS, who were followed up 4 years later with blood samples collected for measuring fasting plasma glucose and haemoglobin A1c. Grip strength (normalized by body weight) and chair‐rising time were assessed at baseline and categorized into tertiles (low, middle, and high groups). Prediabetes at baseline and follow‐up was defined primarily using the American Diabetes Association (ADA) criteria and secondarily using the World Health Organization (WHO) and International Expert Committee (IEC) criteria. Multinomial logistic regression analysis was applied to obtain the odds ratios (ORs) and 95% confidence intervals (CIs).

**Results:**

The mean age of included participants was 59.0 ± 8.6 years, and 46.6% of them were males. During follow‐up, 1646 participants remained as prediabetes, 379 progressed to diabetes, and 598 regressed to normoglycaemia based on ADA criteria. Participants who progressed to diabetes had lower normalized grip strength than those who remained as prediabetes (0.49 ± 0.15 vs. 0.53 ± 0.15, *P* < 0.001), but participants who regressed to normoglycaemia showed the opposite (0.55 ± 0.16 vs. 0.53 ± 0.15, *P* = 0.003). However, chair‐rising time was comparable across different groups (*P*_*overall*_ = 0.17). Compared with participants in low normalized grip strength or high chair‐rising time group, those in high normalized grip strength or low chair‐rising time group had decreased odds of progression to diabetes (OR 0.62, 95% CI 0.44 to 0.87; and OR 0.69, 95% CI 0.51 to 0.93, respectively) after multivariable adjustment. However, both were unrelated to the odds of regression to normoglycaemia (OR 0.94, 95% CI 0.71 to 1.25; and OR 0.84, 95% CI 0.65 to 1.07, respectively). These outcomes remained generally comparable when prediabetes was defined by WHO or IEC criteria. Higher normalized grip strength but not lower chair‐rising time was prospectively associated with lower blood pressure, better glycaemic condition, and lower inflammation (all *P* ≤ 0.04).

**Conclusions:**

High muscle strength is associated with reduced odds of progression to diabetes but does not predict regression to normoglycaemia in prediabetes. Future studies are warranted to assess whether increases in muscle strength promote prediabetes regression.

## Introduction

The prevalence of prediabetes is increased in parallel with that of obesity,^1^, ^2^ and the latest survey shows that prediabetes affects approximately one‐third of the Chinese adults.^1^ Prediabetes is associated with increased risk of cardiovascular disease and all‐cause mortality,^3^, ^4^ and its progression to diabetes may increase the risk further.^5^ However, regression from prediabetes to normoglycaemia may reduce the risk of diabetes^6^ as well as the risk of cardiovascular disease and all‐cause mortality during follow‐up.^5^, ^7^ Identification of modifiable factors to aid in preventing prediabetes progression and/or promoting prediabetes regression is therefore of major clinical importance.

Previous population‐based cohort studies have documented that larger body mass index (BMI) and higher levels of triglycerides (TG) may accelerate the progression of prediabetes to diabetes,^2^, ^4^, ^8^ while reduction in body weight and the use of metformin have the potential to promote its regression to normoglycaemia.^4^, ^9^, ^10^ It is well recognized that skeletal muscle is involved in the maintenance of glycaemic homeostasis upon dynamic uptake and storage of glucose.^11^, ^12^ However, very few studies have explored whether muscle strength, assessed by grip strength and chair‐rising time, plays any role in prediabetes progression or regression. A recent study by Zhang *et al*., which enrolled 328 older Chinese adults with prediabetes, pioneered into this issue, showing that high muscle strength reduced the odds of diabetes over the 3 year follow‐up,^13^ although this study had a relatively small sample size, muscle strength was measured by grip strength only, and prediabetes was defined by only fasting plasma glucose (FPG). It remains unclear as to whether high muscle strength at baseline is associated with prediabetes regression and favourable cardiometabolic control at follow‐up.

Furthermore, there are substantial variations in the definitions of prediabetes based on FPG [5.6–6.9 mmol/L by American Diabetes Association (ADA) and 6.1–6.9 mmol/L by World Health Organization (WHO)^14^] and/or haemoglobin A1c (HbA1c) [5.7–6.4% (39–47 mmol/mol) by ADA and 6.0–6.4% (42–47 mmol/mol) by International Expert Committee (IEC)^15^]. However, no studies have assessed whether these differences may influence the association of muscle strength with prediabetes progression or regression.

By employing FPG‐based and/or HbA1c‐based definitions of prediabetes based on the China Health and Retirement Longitudinal Study (CHARLS) that enrolled middle‐aged and older adults,^16^, ^17^ we conducted this study to address the following questions: (i) is muscle strength related to prediabetes progression or regression? (ii) do different definitions of prediabetes (i.e. ADA, WHO, and IEC criteria) affect the relationships? and (iii) does muscle strength prospectively correlate with cardiometabolic health?

## Methods

### Study population

The CHARLS is a prospective population‐based cohort study, which enrolled a nationally representative sample of community‐dwellers aged ≥45 years from 28 provinces in China. The details about the design of CHARLS were published elsewhere.^16^, ^17^ In this study, data from the 2011–2012 (baseline) and 2015 waves of CHARLS were used, where blood samples were collected. This study was conducted in line with the Declaration of Helsinki, and the protocol of CHARLS was approved by the Ethical Review Committee of Peking University (IRB00001052–11015). All participants provided written informed consent at the time of participation. This study was also reported following the Strengthening the Reporting of Observational Studies in Epidemiology guideline.

**Figure 1:**
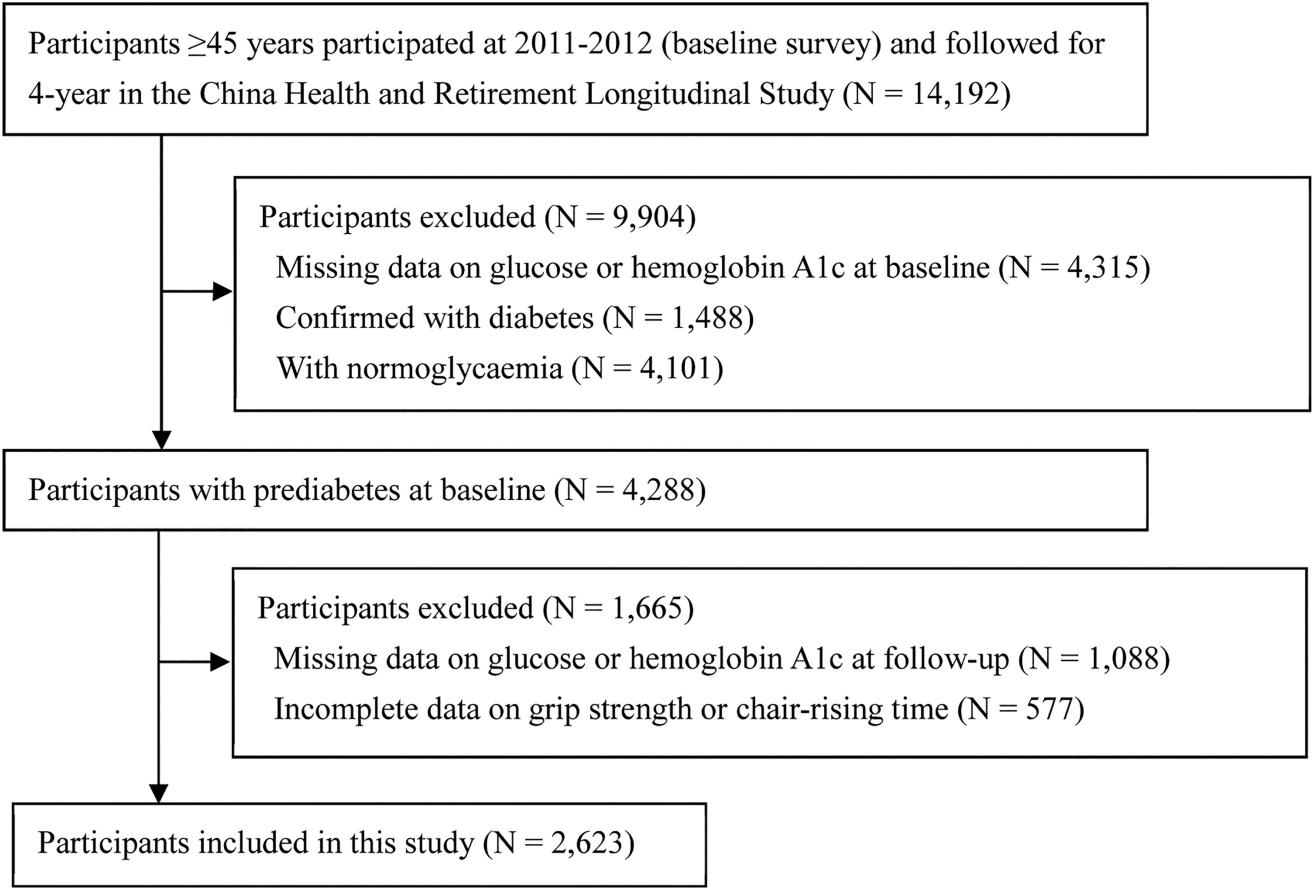
Study flow chart.

Of the 14 192 participants who were followed up in the 2015 wave, we excluded 5403 participants with missing FPG or HbA1c measurements. We also excluded 1488 participants with confirmed diabetes, 4101 with normoglycaemia, and 577 with incomplete data on muscle strength in the 2011–2012 wave, leaving 2623 participants with prediabetes (defined by ADA criteria; see below) eligible for the present analysis (*Figure* 1).

### Data collection

Information on demographic factors (including age and sex), health behaviours (including history of smoking and drinking), and medical history (including hypertension, dyslipidaemia, diabetes, heart disease, and medication use) was collected by well‐trained interviewers (Supporting Information, *Table* *S1*). Anthropometric parameters including body weight, height, and waist circumference (WC) were measured based on standard protocols. BMI (kg/m^2^) was calculated as body weight/(height^2^).^18^ A Body Shape Index (ABSI), which reflects adipose tissue accumulation and is associated with risk of diabetes,^19^, ^20^ is calculated as WC/(BMI^(2/3)^)/(height^(1/2)^). Systolic blood pressure (SBP) and diastolic blood pressure (DBP) at rest were measured three times, and their averages were used. Hypertension was defined as SBP ≥ 140 mmHg, DBP ≥ 90 mmHg, disease history, and/or the use of antihypertensive medications.^21^ Fasting blood samples in each wave were collected for measurements of FPG, HbA1c, total cholesterol (TC), TG, high‐density lipoprotein‐cholesterol (HDL‐c), low‐density lipoprotein‐cholesterol (LDL‐c), uric acid, and high‐sensitivity C‐reactive protein (hs‐CRP). However, a small proportion of blood samples were not fasted, and their glucose was considered random plasma glucose (RPG).^16^ Dyslipidaemia was defined as previously suggested.^21^

### Assessment of muscle strength

Muscle strength, as represented by normalized grip strength and chair‐rising time, was assessed at baseline. Grip strength from the dominant hand was determined using a handgrip dynamometer (YuejianTM WL‐1000 dynamometer) twice, and the average was used. In light of the substantial covariance between grip strength and body weight, grip strength was normalized to body weight [calculated as grip strength (kg)/body weight (kg)],^22^, ^23^ which is termed as normalized grip strength. Chair‐rising time was recorded using a stopwatch by guiding participants to stand up and sit down for five repetitions on a chair at their fastest pace.

### Ascertainment of prediabetes, diabetes, and normoglycaemia

In this study, the classifications of prediabetes, diabetes, and normoglycaemia were based primarily on the ADA criteria and secondarily on the WHO and IEC criteria.

- ADA criteria (primary definition): prediabetes was defined as FPG 5.6–6.9 mmol/L or HbA1c 5.7–6.4% (39–47 mmol/mol); diabetes was as FPG ≥ 7.0 mmol/L, HbA1c ≥ 6.5% (48 mmol/mol), self‐reported history, and/or the use of anti‐diabetic medications; and normoglycaemia was as FPG < 5.6 mmol/L and HbA1c < 5.7% (39 mmol/mol).^24^
- WHO criteria (secondary definition): prediabetes was defined as FPG 6.1–6.9 mmol/L; diabetes as FPG ≥ 7.0 mmol/L, HbA1c ≥ 6.5% (48 mmol/mol), self‐reported history, and/or the use of anti‐diabetic medications; and normoglycaemia as FPG < 6.1 mmol/L.^14^
- IEC criteria (secondary definition): prediabetes was defined as FPG 5.6–6.9 mmol/L and/or HbA1c 6.0–6.4% (42–47 mmol/mol); diabetes as FPG ≥ 7.0 mmol/L, HbA1c ≥ 6.5% (48 mmol/mol), self‐reported history, and/or the use of anti‐diabetic medications; and normoglycaemia as FPG < 5.6 mmol/L or HbA1c < 6.0% (42 mmol/mol).^15^

Moreover, for participants with RPG, they were considered having diabetes if RPG was ≥11.1 mmol/L, and normoglycaemia if RPG < 7.8 mmol/L.

### Statistical analysis

The normality of continuous variables reported in our study (e.g. normalized grip strength) is inspected visually by QQ‐plot, which later showed no serious violations of normality assumptions. Continuous variables in our study are presented as means ± standard deviations and categorical variables as numbers (percentages). Participants were stratified into three groups over follow‐up: (i) progression to diabetes, (ii) regression to normoglycaemia, and (iii) remained as prediabetes. Differences across groups at baseline were compared using one‐way analysis of variance or *χ* ^2^ test, as appropriate, with Bonferroni's correction for multiple comparisons. For participants with missing data on BMI, height, WC, and cardiometabolic markers (including SBP, DBP, TC, TG, HLD‐c, LDL‐c, and hs‐CRP, except FPG and HbA1c) at baseline, they were imputed using the Markov Chain Monte Carlo method.

Multinomial logistic regression analysis was conducted to obtain the odds ratios (ORs) and 95% confidence intervals (CIs) for the association of muscle strength in tertiles with progression to diabetes or regression to normoglycaemia. Three different models were introduced: Model 1, without adjustment; Model 2, adjusted for age and sex; and Model 3, additionally adjusted for ABSI, health behaviours (including history of smoking and drinking), medical history (presence of hypertension, dyslipidaemia, and heart disease), SBP, DBP, TG, TC, LDL‐c, HDL‐c, hs‐CRP, and HbA1c at baseline unless otherwise stated. The variables selected in these models were based on the clinical relevance and the results with significance from the comparisons across groups. Linear regression analysis was used to assess the association between muscle strength and cardiometabolic markers with or without adjustment for age, sex, ABSI, and health behaviours.

In this study, the primary analyses and/or outcomes were derived from participants with prediabetes ascertained by the ADA criteria. However, we also did secondary analyses on participants with prediabetes diagnosed using the WHO or IEC criteria. Subgroup analyses stratified by age (≥60 vs. <60 years) and sex (male vs. female) were performed. Sensitivity analyses by excluding participants without fasting blood samples or those with incomplete data were also conducted separately. Because recent studies have used BMI‐normalized grip strength instead of body weight to account for the contribution of height,^25^, ^26^ we performed a supplemental analysis to assess whether this would affect our main findings. All analyses were performed using Stata (Version 14.0, StataCorp LP, College Station, TX, USA), and *P* values < 0.05 were considered significant.

## Results

### Baseline characteristics

Of the included 2623 participants with prediabetes (mean age 59.0 ± 8.6 years, 46.6% males) based on the ADA criteria, 1646 remained as prediabetes, 379 progressed to diabetes, and 598 regressed to normoglycaemia during the 4 year follow‐up. Their baseline characteristics are shown in *Table* 1. Compared with participants who remained as prediabetes, those who progressed to diabetes had higher prevalence of hypertension and dyslipidaemia and showed higher BMI, SBP, DBP, TG, hs‐CRP, FPG, and HbA1c (all *P* < 0.01). In contrast, participants who regressed to normoglycaemia were younger and showed lower TC, LDL‐c, hs‐CRP, FPG, and HbA1c (all *P* < 0.01). Moreover, participants who progressed to diabetes had lower normalized grip strength than those who remained as prediabetes (0.49 ± 0.15 vs. 0.53 ± 0.15, *P* < 0.001), but participants who regressed to normoglycaemia showed the opposite (0.55 ± 0.16 vs. 0.53 ± 0.15, *P* = 0.003). However, chair‐rising time was comparable across different groups (*P*_*overall*_ = 0.17).

**Table 1:** Baseline characteristics of participants stratified by glycaemic condition^a^

	Total	Remained as prediabetes	Regression to normoglycaemia	Progression to diabetes	*P*_*overall*_
Sample size (*n*)	2623	1646	598	379	
Age (years)	59.0 ± 8.6	59.2 ± 8.5	57.5 ± 8.5*	60.3 ± 8.7	<0.001
Male (%)	1220 (46.6%)	736 (44.8%)	322 (53.8%)*	162 (42.7%)	<0.001
Smoking (%)^b^ ^,^ ^c^	1006 (38.4%)	613 (37.2%)	247 (41.3%)	146 (38.5%)	0.20
Drinking (%)^c^	866 (33.0%)	544 (33.0%)	217 (36.3%)	105 (27.7%)	0.02
Disease history^c^					
Hypertension (%)	1119 (42.7%)	682 (41.4%)	231 (38.6%)	206 (54.4%)^#^	<0.001
Dyslipidaemia (%)	1186 (45.2%)	730 (44.3%)	250 (41.8%)	206 (54.4%)^#^	<0.001
Heart disease (%)^b^	310 (11.9%)	191 (11.7%)	60 (10.1%)	59 (15.8%)	0.03
BMI (kg/m^2^)	23.8 ± 4.0	23.6 ± 3.8	23.5 ± 4.2	25.3 ± 4.2^#^	<0.001
ABSI	0.071 ± 0.008	0.071 ± 0.008	0.069 ± 0.008	0.071 ± 0.007	0.02
SBP (mmHg)	131.4 ± 25.1	131.0 ± 21.1	130.4 ± 30.8	134.5 ± 30.4^#^	0.03
DBP (mmHg)	76.2 ± 13.1	75.8 ± 11.8	76.2 ± 15.2	77.8 ± 15.1^#^	0.04
TC (mg/dL)	197.1 ± 38.3	199.4 ± 38.4	189.0 ± 37.0*	200.0 ± 38.4	<0.001
TG (mg/dL)	133.1 ± 85.4	131.8 ± 82.9	130.0 ± 92.1	143.8 ± 84.9^#^	0.03
HDL‐c (mg/dL)	51.3 ± 15.3	52.0 ± 15.6	51.3 ± 14.9	48.2 ± 14.1^#^	<0.001
LDL‐c (mg/dL)	119.6 ± 35.6	121.6 ± 36.0	112.3 ± 34.4*	122.7 ± 33.8	<0.001
UA (mg/dL)	4.45 ± 1.22	4.45 ± 1.21	4.42 ± 1.23	4.52 ± 1.25	0.45
log(hs‐CRP) (mg/L)	0.15 ± 1.04	0.14 ± 1.03	0.02 ± 0.99*	0.40 ± 1.10^#^	<0.001
FPG (mg/dL)^b^	107.5 ± 7.1	107.4 ± 7.1	106.5 ± 6.3*	109.7 ± 7.5^#^	<0.001
HbA1c (%)	5.2 ± 0.4	5.2 ± 0.4	5.0 ± 0.3*	5.4 ± 0.4^#^	<0.001
HbA1c (mol/mmol)	33 ± 4.4	33 ± 4.4	31 ± 3.3*	36 ± 4.4^#^	<0.001
Normalized grip strength	0.53 ± 0.16	0.53 ± 0.15	0.55 ± 0.16*	0.49 ± 0.15^#^	<0.001
Chair‐rising time (s)	10.7 ± 3.9	10.7 ± 4.1	10.5 ± 3.4	11.0 ± 3.8	0.17

ABSI, A Body Shape Index; BMI, body mass index; DBP, diastolic blood pressure; FPG, fasting plasma glucose; HbA1c, haemoglobin A1c; HDL‐c, high‐density lipoprotein‐cholesterol; hs‐CRP, high‐sensitivity C‐reactive protein; LDL‐c, low‐density lipoprotein‐cholesterol; SBP, systolic blood pressure; TC, total cholesterol; TG, triglycerides; UA, uric acid.

^a^ They were compared using one‐way analysis of variance or *χ* ^2^ test, as appropriate, with Bonferroni's correction for multiple comparisons.

^b^ There were 2 and 19 participants with missing information for history of smoking and heart disease, respectively; and 37 participants without fasting blood samples at baseline.

^c^ History was obtained by questionnaires with answers of yes or no in general.

* *P* < 0.01, compared between regression to normoglycaemia vs. remained as prediabetes.

^#^ *P* < 0.01, compared between progression to diabetes vs. remained as prediabetes.

### Normalized grip strength and prediabetes progression and regression

The association of normalized grip strength with prediabetes progression or regression is shown in *Table* 2. Based on the ADA criteria, participants with high normalized grip strength (>0.59) exhibited decreased odds of progression to diabetes (OR 0.54, 95% CI 0.40 to 0.71), and increased odds of regression to normoglycaemia (OR 1.31, 95% CI 1.04 to 1.66), when compared with participants with low normalized grip strength (<0.46), in the unadjusted model (Model 1). The association of normalized grip strength with the odds of progression to diabetes, but not the odds of regression to normoglycaemia, remained significant after controlling for multivariable (OR 0.62, 95% CI 0.44 to 0.87, Model 3). Moreover, the analysis on grip strength normalized by BMI (*Table* *S2*), as opposed to normalized by body weight (*Table* 2), showed similar outcomes. When prediabetes was defined by the WHO or IEC criteria, the results were comparable with those based on the ADA criteria in the multivariable‐adjusted model (Model 3).

**Table 2:** Normalized grip strength and prediabetes regression and progression

Variables	No. of cases/total	Model 1^a^	Model 2^b^	Model 3^c^
		OR (95% CIs)	OR (95% CIs)	OR (95% CIs)
**ADA criteria for prediabetes, diabetes, and normoglycaemia (primary analysis)**				
Prediabetes progression				
Low (Tertile 1, <0.46)	165/875	1 (Ref.)	1 (Ref.)	1 (Ref.)
Middle (Tertile 2, 0.46–0.59)	123/874	0.75 (0.57 to 0.97)	0.72 (0.55 to 0.95)	0.85 (0.64 to 1.13)
High (Tertile 3, >0.59)	91/874	0.54 (0.40 to 0.71)	0.49 (0.36 to 0.68)	0.62 (0.44 to 0.87)
*P* for trend		<0.001	<0.001	0.007
Prediabetes regression				
Low (Tertile 1, <0.46)	166/875	1 (Ref.)	1 (Ref.)	1 (Ref.)
Middle (Tertile 2, 0.46–0.59)	208/874	1.26 (0.99 to 1.59)	1.10 (0.86 to 1.40)	1.03 (0.80 to 1.32)
High (Tertile 3, >0.59)	224/874	1.31 (1.04 to 1.66)	0.97 (0.74 to 1.27)	0.94 (0.71 to 1.25)
*P* for trend		0.02	0.80	0.67
**WHO criteria for prediabetes, diabetes, and normoglycaemia (secondary analysis)**				
Prediabetes progression				
Low (Tertile 1, <0.45)	75/317	1 (Ref.)	1 (Ref.)	1 (Ref.)
Middle (Tertile 2, 0.45–0.59)	69/317	0.79 (0.42 to 1.49)	0.74 (0.38 to 1.42)	0.79 (0.76 to 1.78)
High (Tertile 3, >0.59)	40/316	0.38 (0.20 to 0.72)	0.32 (0.15 to 0.67)	0.56 (0.33 to 0.95)
Prediabetes regression				
Low (Tertile 1, <0.45)	218/317	1 (Ref.)	1 (Ref.)	1 (Ref.)
Middle (Tertile 2, 0.45–0.59)	220/317	0.87 (0.49 to 1.54)	0.81 (0.45 to 1.47)	1.46 (0.79 to 2.71)
High (Tertile 3, >0.59)	242/316	0.78 (0.45 to 1.36)	0.68 (0.36 to 1.29)	1.78 (0.90 to 3.51)
**IEC criteria for prediabetes, diabetes, and normoglycaemia (secondary analysis)**				
Prediabetes progression				
Low (Tertile 1, <0.46)	158/849	1 (Ref.)	1 (Ref.)	1 (Ref.)
Middle (Tertile 2, 0.46–0.59)	121/849	0.84 (0.64 to 1.17)	0.82 (0.61 to 1.09)	0.92 (0.68 to 1.25)
High (Tertile 3, >0.59)	83/848	0.58 (0.43 to 0.79)	0.53 (0.38 to 0.76)	0.65 (0.45 to 0.94)
Prediabetes regression				
Low (Tertile 1, <0.46)	317/849	1 (Ref.)	1 (Ref.)	1 (Ref.)
Middle (Tertile 2, 0.46–0.59)	389/849	1.35 (1.10 to 1.67)	1.25 (1.01 to 1.55)	1.14 (0.92 to 1.43)
High (Tertile 3, >0.59)	429/848	1.50 (1.22 to 1.85)	1.27 (0.99 to 1.61)	1.16 (0.90 to 1.49)

ADA, American Diabetes Association; CIs, confidence intervals; IEC, International Expert Committee; OR, odds ratio; WHO, World Health Organization.

^a^ Unadjusted.

^b^ Adjusted for age and sex.

^c^ Adjusted for age, sex, A Body Shape Index, history of smoking and drinking, presence of hypertension, dyslipidaemia, and heart disease, systolic blood pressure, diastolic blood pressure, triglycerides, total cholesterol, low‐density lipoprotein‐cholesterol, high‐density lipoprotein‐cholesterol, high‐sensitivity C‐reactive protein, and haemoglobin A1c at baseline.

Sensitivity analyses upon the exclusion of participants without fasting blood samples or those with imputed data on cardiometabolic markers showed that they did not influence the primary outcomes (*Table* *S3*). Subgroup analysis suggested a comparable association of normalized grip strength with odds of progression to diabetes in older adults ≥60 versus <60 years (*P*_*interaction*_ = 0.71) or in males versus females (*P*_*interaction*_ = 0.28, *Figure* *S1*).

### Chair‐rising time and prediabetes progression and regression

The association of chair‐rising time with prediabetes progression and regression based on the ADA criteria is shown in *Table* 3. Compared with participants with high chair‐rising time (>11.5 s), those with low chair‐rising time (<8.8 s) had reduced odds of progression to diabetes in the unadjusted model (OR 0.66, 95% CI 0.50 to 0.87, Model 1) and the multivariable‐adjusted model (OR 0.69, 95% CI 0.51 to 0.93, Model 3). However, low chair‐rising time was not related to increased odds of regression to normoglycaemia (*Table* 3). When prediabetes was defined by the WHO or IEC criteria, the results remained generally comparable with those based on the ADA criteria.

**Table 3:** Chair‐rising time and prediabetes regression and progression

Variables	No. of cases/total	Model 1^a^	Model 2^b^	Model 3^c^
		OR (95% CIs)	OR (95% CIs)	OR (95% CIs)
**ADA criteria for prediabetes, diabetes, and normoglycaemia (primary analysis)**				
Prediabetes progression				
Low (Tertile 1, <8.8 s)	101/875	0.66 (0.50 to 0.87)	0.70 (0.52 to 0.94)	0.69 (0.51 to 0.93)
Middle (Tertile 2, 8.8–11.5 s)	134/878	0.89 (0.69 to 1.16)	0.92 (0.70 to 1.20)	0.90 (0.69 to 1.20)
High (Tertile 3, >11.5 s)	144/870	1 (Ref.)	1 (Ref.)	1 (Ref.)
*P* for trend		0.004	0.02	0.02
Prediabetes regression				
Low (Tertile 1, <8.8 s)	210/875	1.01 (0.80 to 1.26)	0.82 (0.65 to 1.04)	0.84 (0.65 to 1.07)
Middle (Tertile 2, 8.8–11.5 s)	192/878	0.94 (0.75 to 1.19)	0.86 (0.68 to 1.08)	0.87 (0.68 to 1.11)
High (Tertile 3, >11.5 s)	196/870	1 (Ref.)	1 (Ref.)	1 (Ref.)
*P* for trend		0.94	0.11	0.16
**WHO criteria for prediabetes, diabetes, and normoglycaemia (secondary analysis)**				
Prediabetes progression				
Low (Tertile 1, <8.8 s)	50/320	0.60 (0.31 to 1.16)	0.65 (0.33 to 1.30)	0.63 (0.31 to 1.30)
Middle (Tertile 2, 8.8–11.5 s)	63/314	0.57 (0.30 to 1.06)	0.59 (0.31 to 1.10)	0.55 (0.29 to 1.07)
High (Tertile 3, >11.5 s)	71/316	1 (Ref.)	1 (Ref.)	1 (Ref.)
Prediabetes regression				
Low (Tertile 1, <8.8 s)	243/320	0.93 (0.52 to 1.67)	0.92 (0.50 to 1.68)	1.02 (0.54 to 1.93)
Middle (Tertile 2, 8.8–11.5 s)	215/314	0.62 (0.35 to 1.08)	0.61 (0.35 to 1.08)	0.62 (0.34 to 1.10)
High (Tertile 3, >11.5 s)	222/316	1 (Ref.)	1 (Ref.)	1 (Ref.)
**IEC criteria for prediabetes, diabetes, and normoglycaemia (secondary analysis)**				
Prediabetes progression				
Low (Tertile 1, <8.8 s)	94/850	0.63 (0.47 to 0.85)	0.65 (0.48 to 0.89)	0.64 (0.46 to 0.88)
Middle (Tertile 2, 8.8–11.5 s)	128/848	0.90 (0.68 to 1.19)	0.91 (0.68 to 1.21)	0.89 (0.66 to 1.20)
High (Tertile 3, >11.5 s)	140/848	1 (Ref.)	1 (Ref.)	1 (Ref.)
Prediabetes regression				
Low (Tertile 1, <8.8 s)	394/850	1.01 (0.82 to 1.23)	0.85 (0.68 to 1.05)	0.86 (0.69 to 1.08)
Middle (Tertile 2, 8.8–11.5 s)	373/848	0.99 (0.81 to 1.22)	0.92 (0.74 to 1.13)	0.93 (0.74 to 1.15)
High (Tertile 3, >11.5 s)	368/848	1 (Ref.)	1 (Ref.)	1 (Ref.)

ADA, American Diabetes Association; CIs, confidence intervals; IEC, International Expert Committee; OR, odds ratio; WHO, World Health Organization.

^a^ Unadjusted.

^b^ Adjusted for age and sex.

^c^ Adjusted for age, sex, A Body Shape Index, history of smoking and drinking, presence of hypertension, dyslipidaemia, and heart disease, systolic blood pressure, diastolic blood pressure, triglycerides, total cholesterol, low‐density lipoprotein‐cholesterol, high‐density lipoprotein‐cholesterol, high‐sensitivity C‐reactive protein, and haemoglobin A1c at baseline.

Sensitivity analyses after excluding participants without fasting blood samples or those with incomplete on cardiometabolic markers showed similar results as the primary ones (*Table* *S4*). The association of chair‐rising time with odds of progression to diabetes was comparable in older adults ≥60 versus <60 years (*P*_*interaction*_ = 0.24) or in males versus females (*P*_*interaction*_ = 0.26, *Figure* *S2*).

### Muscle strength and cardiometabolic health at follow‐up

Linear regression analysis showed that high normalized grip strength at baseline was prospectively associated with high levels of HDL‐c (*P* < 0.001) but with low levels of DBP, FPG, HbA1c, TG, and hs‐CRP at 4 year follow‐up after multivariable adjustment (all *P* ≤ 0.04, *Table* 4). Yet high chair‐rising time at baseline was only prospectively associated with low levels of HDL‐c (*Sβ* = −0.06, *P* = 0.002, *Table* 5).

**Table 4:** Prospective association of normalized grip strength at baseline with cardiometabolic health at follow‐up

Parameters	Simple linear regression analysis		Multivariable linear regression analysis^a^	
	*Sβ*	*P*	*Sβ′*	*P*
Blood pressure				
SBP (*n* = 2553)	−0.09	<0.001	−0.02	0.19
DBP (*n* = 2550)	−0.04	0.03	−0.04	0.02
Glycaemic control				
FPG (*n* = 2256)	−0.12	<0.001	−0.04	0.04
HbA1c (*n* = 2623)	−0.13	<0.001	−0.04	0.02
Lipid profiles				
TC (*n* = 2619)	−0.09	<0.001	0.03	0.06
TG (*n* = 2619)	−0.15	<0.001	−0.05	0.003
HDL‐c (*n* = 2619)	0.08	<0.001	0.07	<0.001
LDL‐c (*n* = 2618)	−0.07	<0.001	0.02	0.15
Inflammation				
hs‐CRP (*n* = 2618)	−0.15	<0.001	−0.08	<0.001

DBP, diastolic blood pressure; FPG, fasting plasma glucose; HbA1c, haemoglobin A1c; HDL‐c, high‐density lipoprotein‐cholesterol; hs‐CRP, high‐sensitivity C‐reactive protein; LDL‐c, low‐density lipoprotein‐cholesterol; SBP, systolic blood pressure; *Sβ*, standardized regression coefficient; TC, total cholesterol; TG, triglycerides.

^a^ It was adjusted for age, sex, A Body Shape Index, and history of smoking and drinking.

**Table 5:** Prospective association of normalized chair‐rising time at baseline with cardiometabolic health at follow‐up

Parameters	Simple linear regression analysis		Multivariable linear regression analysis^a^	
	*Sβ*	*P*	*Sβ′*	*P*
Blood pressure				
SBP (*n* = 2553)	0.07	0.001	0.02	0.35
DBP (*n* = 2550)	−0.03	0.17	0.008	0.70
Glycaemic control				
FPG (*n* = 2256)	0.02	0.24	0.001	0.98
HbA1c (*n* = 2623)	0.03	0.19	−0.009	0.65
Lipid profiles				
TC (*n* = 2619)	0.004	0.84	−0.02	0.30
TG (*n* = 2619)	0.005	0.80	0.02	0.34
HDL‐c (*n* = 2619)	−0.06	0.003	−0.06	0.002
LDL‐c (*n* = 2618)	0.02	0.31	−0.008	0.68
Inflammation				
hs‐CRP (*n* = 2618)	0.02	0.43	−0.006	0.77

DBP, diastolic blood pressure; FPG, fasting plasma glucose; HbA1c, haemoglobin A1c; HDL‐c, high‐density lipoprotein‐cholesterol; hs‐CRP, high‐sensitivity C‐reactive protein; LDL‐c, low‐density lipoprotein‐cholesterol; SBP, systolic blood pressure; *Sβ*, standardized regression coefficient; TC, total cholesterol; TG, triglycerides.

^a^ It was adjusted for age, sex, A Body Shape Index, and history of smoking and drinking.

## Discussion

### Summary of main findings

Our study showed for the first time in middle‐aged and older Chinese adults with prediabetes that (i) high muscle strength, as represented by high normalized grip strength and low chair‐rising time, was associated with reduced odds of progression to diabetes during the 4 year follow‐up, although it was not related to an increased probability of regression to normoglycaemia; (ii) these relationships were not affected by the variations in the definitions of prediabetes (i.e. ADA, WHO, or IEC criteria); and (iii) higher normalized grip strength rather than lower chair‐rising time was prospectively associated with better cardiometabolic outcomes on blood pressure, glycaemic indices, and inflammation.

### Interpretations

Previous studies have suggested that low BMI, TG, and HbA1c at baseline as well as the use of metformin may reduce the risk of progression of prediabetes to diabetes.^2^, ^4^, ^8^ Extending to these insights, our observations suggest that large muscle strength, represented by high normalized grip strength or low chair‐rising time, may be effective to prevent prediabetes progression, independently of aforementioned factors such as BMI, TG, and HbA1c. Compared with prediabetes participants with normalized grip strength < 0.46 for the dominant hand or chair‐rising time > 11.5 s for five repetitions of stand‐up and sit‐down, those with normalized grip strength > 0.59 or chair‐rising time < 8.8 s exhibited 38% and 31% lower odds of progression to diabetes, respectively. Considering that high muscle strength is associated with reduced risk of mortality,^27^ our results may add another piece of evidence to partially support the importance of increasing muscle strength for health and well‐being.

Our study confirmed the results reported by Zhang *et al*. that low muscle strength assessed by normalized grip strength was related to reduced odds of prediabetes progression based on 328 older adults with prediabetes defined by FPG during 3 year follow‐up.^13^ However, our study had a substantially larger sample size (approximately 7 times bigger), longer duration of follow‐up (1 more year), and more robust assessment of muscle strength and characterization of prediabetes.

Of note, the association of high normalized grip strength (but not low chair‐rising time) with a high probability of regression to normoglycaemia in prediabetes was not retained after controlling for variables such as age, TG, HbA1c, and hs‐CRP at baseline. This indicates that the contribution of muscle strength to prediabetes regression might be largely influenced by a mixture of other factors at baseline. However, this does not necessarily mean that improvements in normalized grip strength and/or chair‐rising time do not promote prediabetes regression, because there is evidence that an increase in exercise, which generally coincides with an improvement in muscle strength,^28^ was associated with higher odds of regression.^10^

There are substantial variations in the definition of prediabetes, in particular for the cut‐offs of HbA1c and/or FPG,^14^, ^29^ in the current diabetes management guidelines (e.g. in China, HbA1c has not been recommended for defining prediabetes^30^), which may influence the interpretation on the consequences of prediabetes. In the Atherosclerosis Risk in Communities study, Warren *et al*. showed that prediabetes defined using the ADA cut‐off of HbA1c had a lower specificity than the IEC cut‐off in discriminating outcomes such as incident diabetes, cardiovascular disease, and all‐cause mortality.^14^ The authors also showed that the use of HbA1c by any cut‐off from ADA or IEC performed better in risk discrimination for clinical complications than FPG, but the use of ADA recommended FPG cut‐off might be more sensitive.^14^ However, in the current study, we found that such differences in the definitions of prediabetes did not significantly affect the association of muscle strength with prediabetes progression or regression, despite the rates of progression or regression differed from each other, particularly for the outcomes based on the WHO criteria (*Tables* 2 and 3).

The mechanism underling the association of large muscle strength with reduced odds of progression to diabetes in middle‐aged and older adults with prediabetes is not fully understood. As an attempt, our regression analyses provide some insights on this issue, showing that higher normalized grip strength was prospectively related to better cardiometabolic outcomes on blood pressure, glycaemic condition (assessed by FPG and HbA1c), and inflammation (represented by hs‐CRP) and that lower chair‐rising time was correlated with higher HDL levels. Moreover, large muscle strength is correlated with high cardiorespiratory fitness in adults,^28^ the latter of which is also associated with reduced risk of diabetes.^31^ In addition, our regression analyses suggest that normalized grip strength might outperform chair‐rising time in predicting future cardiometabolic health. This supports the use of grip strength for assessment of muscle strength in clinical practice.

### Strengths and limitations

The strengths of this study include a prospective cohort design based on a community‐based population with a large sample size, a rigorous assessment of muscle strength using standardized protocols, as well as the comparison of the influence of different definitions of prediabetes on the study outcomes that generated robust results.

However, several limitations should be considered. First, despite FPG and HbA1c had been used to define prediabetes, the lack of 2 h postprandial glucose data from oral glucose tolerance test may bias the results reported (e.g. failure to capture some participants with prediabetes at baseline^32^). Second, the classifications of prediabetes and diabetes were based on single measurements of FPG and HbA1c, whereas in clinical routine, they might be repeated, particularly in the absence of symptoms. Third, there existed a small proportion of participants with incomplete data on covariates (e.g. WC, SBP, and DBP) at baseline, which were later imputed using the Markov Chain Monte Carlo method. However, sensitivity analysis by excluding these participants revealed comparable results. Fourth, although our analyses have controlled for multivariable, residual confounding from unmeasured factors (e.g. cardiorespiratory fitness and/or physical activity,^31^ carbohydrate intake,^33^ cell adhesion molecules,^34^ or other indicators of body composition such as body fat percentage, fat free mass,^35^ or fat‐to‐muscle mass ratio^36^) cannot be excluded. Fifth, our study showed similar results from the analyses between grip strength normalized by body weight and BMI, yet it remains to be investigated whether grip strength normalized by other measures such as fat free mass or muscle mass would yield different outcomes. Finally, our study included only middle‐aged and older Chinese adults, the conclusion of which might not be generalizable to other populations (e.g. youngsters and adults from other countries).

## Conclusions

In conclusion, high muscle strength was associated with reduced odds of progression to diabetes but not an increased probability of regression to normoglycaemia in middle‐aged and older Chinese adults with prediabetes, independently of the various definitions of prediabetes by different associations/organizations. High muscle strength also prospectively correlated with favourable cardiometabolic control in prediabetes. These, taken together, indicate that increases in muscle strength may benefit future health outcomes in middle‐aged and older adults with prediabetes, although it remains to be explored whether such increases may promote prediabetes regression.

## Conflict of interest

Shanhu Qiu, Xue Cai, Yang Yuan, Bo Xie, Zilin Sun, Duolao Wang, and Tongzhi Wu declare that they have no conflict of interest.

## Funding

This work was partly supported by the National Key R&D Program of China (Grant No. 2016YFC1305700) and the Guangdong Basic and Applied Basic Research Foundation (Grant No. 2020A1515111021). Tongzhi Wu has been supported by a Mid‐Career Fellowship from The Hospital Research Foundation. The funders had no roles in the design of the study and collection, analysis, and interpretation of data or in writing the manuscript.

## Supporting information


**Table S1.** Questionnaires used for obtaining information on health behaviors and medical history.
**Table S2.** Normalized grip strength by body mass index and prediabetes regression and progression.
**Table S3.** Sensitivity analysis for normalized grip strength and prediabetes regression and progression^a^.
**Table S4.** Sensitivity analysis for chair‐rising time and prediabetes regression and progression^a^.
**Figure S1.** Subgroup analyses for the association of normalized grip strength with prediabetes regression and progression.
**Figure S2.** Subgroup analyses for the association of chair‐rising time with prediabetes regression and progression.
