# Supplementary material for: Muscle strength and prediabetes progression and regression in middle‐aged and older adults: a prospective cohort study
Source: J Cachexia Sarcopenia Muscle. 2022 Jan 23;13(2):909–18. doi: 10.1002/jcsm.12905 (PMC8978008; doi:10.1002/jcsm.12905)
Supplement: Supplementary file 1 — Table S1. Questionnaires used for obtaining information on health behaviors and medical history. Table S2. Normalized grip strength by body mass index and prediabetes regression and progression. Table S3. Sensitivity analysis for normalized grip strength and prediabetes regression and progressiona. Table S4. Sensitivity analysis for chair‐rising time and prediabetes regression and progressiona. Figure S1. Subgroup analyses for the association of normalized grip strength with prediabetes regression and progression. Figure S2. Subgroup analyses for the association of chair‐rising time with prediabetes regression and progression. [file JCSM-13-909-s001.docx]

# Supporting Information

# Supporting Information Table S1. Questionnaires used for obtaining information on health behaviors and medical history.

| Information | Questionnaires used | Answers |
| --- | --- | --- |
| Health behaviors |  |  |
| History of smoking | Have you ever chewed tobacco, smoked a pipe, smoked self-rolled cigarettes, or smoked cigarettes/cigars? | (i) Yes  (ii) No |
| History of drinking^a^ | Did you drink any alcoholic beverages, such as beer, wine, or liquor in the past year? | (i) Drink more than once a month.  (ii) Drink but less than once a month  (iii) None of these |
| Medical history |  |  |
| Hypertension | Have you been diagnosed with “Hypertension” by a doctor? | (i) Yes  (ii) No |
| Dyslipidemia | Have you been diagnosed with “Dyslipidemia” by a doctor? | (i) Yes  (ii) No |
| Diabetes | Are you now taking any of the following treatments to treat or control your diabetes? (Taking Chinese traditional medicine, taking Western modern medicine, taking insulin injections, and none of above) | Participants with answers on this question were considered with diabetes, otherwise were not. |
| Heart disease | Have you been diagnosed with “Heart attack, coronary heart disease, angina, congestive heart failure, or other heart problems” by a doctor? | (i) Yes  (ii) No |

^a^ Answers of (i) and (ii) were categorized as “Yes”, and (iii) as “No”.

# Supporting Information Table S2. Normalized grip strength by body mass index and prediabetes regression and progression.

| Variables (unit: kg/(kg/m^2^)) | No. of Cases/total | Model 1^a^ | Model 2^b^ | Model 3^c^ |
| --- | --- | --- | --- | --- |
|  |  | OR (95% CIs) | OR (95% CIs) | OR (95% CIs) |
| **ADA criteria for prediabetes, diabetes, and normoglycemia** | | | |  |
| Prediabetes progression |  |  |  |  |
| Low (Tertile 1, <1.10) | 163/872 | 1 (Ref.) | 1 (Ref.) | 1 (Ref.) |
| Middle (Tertile 2, 1.10 – 1.52) | 123/872 | 0.72 (0.60 to 1.02) | 0.72 (0.55 to 0.96) | 0.89 (0.66 to 1.20) |
| High (Tertile 3, >1.52) | 93/872 | 0.57 (0.43 to 0.76) | 0.47 (0.32 to 0.67) | 0.65 (0.44 to 0.97) |
| Prediabetes regression | |  |  |  |
| Low (Tertile 1, <1.10) | 155/875 | 1 (Ref.) | 1 (Ref.) | 1 (Ref.) |
| Middle (Tertile 2, 1.10 – 1.52) | 216/874 | 1.44 (1.14 to 1.83) | 1.23 (0.96 to 1.60) | 1.18 (0.91 to 1.54) |
| High (Tertile 3, >1.52) | 227/874 | 1.48 (1.17 to 1.88) | 1.00 (0.74 to 1.37) | 0.97 (0.70 to 1.34) |

OR, odds ratio; CI, confidence interval; ADA, American Diabetes Association

^a^ Unadjusted.

^b^ Adjusted for age and sex.

^c^ Adjusted for age, sex, a body shape index, history of smoking and drinking, presence of hypertension, dyslipidemia, and heart disease, systolic blood pressure, diastolic blood pressure, triglycerides, total cholesterol, low-density lipoprotein-cholesterol, high-density lipoprotein-cholesterol, high-sensitivity C-reactive protein, and hemoglobin A1c at baseline.

# Supporting Information Table S3. Sensitivity analysis for normalized grip strength and prediabetes regression and progression^a^.

| Variables | No. of Cases/total | Model 1^b^ | Model 2^c^ | Model 3^d^ |
| --- | --- | --- | --- | --- |
|  |  | OR (95% CIs) | OR (95% CIs) | OR (95% CIs) |
| **Excluding participants without fasting samples** | | | |  |
| Prediabetes progression | |  |  |  |
| Low (Tertile 1) | 146/742 | 1 (Ref.) | 1 (Ref.) | 1 (Ref.) |
| Middle (Tertile 2) | 108/742 | 0.74 (0.56 to 0.99) | 0.72 (0.54 to 0.96) | 0.85 (0.62 to 1.15) |
| High (Tertile 3) | 77/741 | 0.52 (0.38 to 0.70) | 0.47 (0.33 to 0.67) | 0.60 (0.41 to 0.86) |
| Prediabetes regression | |  |  |  |
| Low (Tertile 1) | 134/742 | 1 (Ref.) | 1 (Ref.) | 1 (Ref.) |
| Middle (Tertile 2) | 175/742 | 1.31 (1.01 to 1.70) | 1.16 (0.89 to 1.52) | 1.10 (0.83 to 1.45) |
| High (Tertile 3) | 191/741 | 1.39 (1.08 to 1.80) | 1.05 (0.78 to 1.41) | 1.03 (0.75 to 1.41) |
| **Excluding participants with incomplete data^e^** | | | |  |
| Prediabetes progression | |  |  |  |
| Low (Tertile 1) | 161/859 | 1 (Ref.) | 1 (Ref.) | 1 (Ref.) |
| Middle (Tertile 2) | 121/859 | 0.75 (0.58 to 0.98) | 0.74 (0.56 to 0.97) | 0.87 (0.65 to 1.15) |
| High (Tertile 3) | 88/859 | 0.53 (0.40 to 0.71) | 0.50 (0.36 to 0.69) | 0.62 (0.44 to 0.89) |
| Prediabetes regression | |  |  |  |
| Low (Tertile 1) | 162/860 | 1 (Ref.) | 1 (Ref.) | 1 (Ref.) |
| Middle (Tertile 2) | 203/859 | 1.26 (0.99 to 1.59) | 1.09 (0.85 to 1.40) | 1.03 (0.79 to 1.32) |
| High (Tertile 3) | 221/859 | 1.33 (1.05 to 1.68) | 0.98 (0.74 to 1.29) | 0.92 (0.69 to 1.23) |

OR, odds ratio; CI, confidence interval

^a^ The definitions of prediabetes, diabetes, and normoglycemia were based on the American Diabetes Association criteria.

^b^ Unadjusted.

^c^ Adjusted for age and sex.

^d^ Adjusted for age, sex, a body shape index, history of smoking and drinking, presence of hypertension, dyslipidemia, and heart disease, systolic blood pressure, diastolic blood pressure, triglycerides, total cholesterol, low-density lipoprotein-cholesterol, high-density lipoprotein-cholesterol, high-sensitivity C-reactive protein, and hemoglobin A1c at baseline.

^e^ Incomplete data mainly included age, body mass index, waist circumference, blood pressure, triglycerides, total cholesterol, high-density lipoprotein-cholesterol, and low-density lipoprotein-cholesterol.

# Supporting Information Table S4. Sensitivity analysis for chair-rising time and prediabetes regression and progression^a^.

| Variables | No. of Cases/Total | Model 1^b^ | Model 2^c^ | Model 3^d^ |
| --- | --- | --- | --- | --- |
|  |  | OR (95% CIs) | OR (95% CIs) | OR (95% CIs) |
| **Excluding participants without fasting samples** | | | |  |
| Prediabetes progression | |  |  |  |
| Low (Tertile 1) | 88/744 | 0.63 (0.47 to 0.85) | 0.69 (0.50 to 0.93) | 0.67 (0.49 to 0.93) |
| Middle (Tertile 2) | 113/742 | 0.83 (0.62 to 1.10) | 0.86 (0.64 to 1.14) | 0.84 (0.63 to 1.14) |
| High (Tertile 3) | 130/739 | 1 (Ref.) | 1 (Ref.) | 1 (Ref.) |
| Prediabetes regression | |  |  |  |
| Low (Tertile 1) | 178/744 | 1.03 (0.80 to 1.32) | 0.83 (0.64 to 1.08) | 0.85 (0.65 to 1.11) |
| Middle (Tertile 2) | 160/742 | 0.94 (0.73 to 1.21) | 0.86 (0.66 to 1.11) | 0.86 (0.66 to 1.12) |
| High (Tertile 3) | 162/739 | 1 (Ref.) | 1 (Ref.) | 1 (Ref.) |
| **Excluding participants with incomplete data^e^** | | | |  |
| Prediabetes progression | |  |  |  |
| Low (Tertile 1) | 98/860 | 0.64 (0.49 to 0.86) | 0.69 (0.51 to 0.92) | 0.68 (0.50 to 0.92) |
| Middle (Tertile 2) | 129/858 | 0.87 (0.67 to 1.14) | 0.90 (0.69 to 1.17) | 0.89 (0.67 to 1.17) |
| High (Tertile 3) | 143/859 | 1 (Ref.) | 1 (Ref.) | 1 (Ref.) |
| Prediabetes regression | |  |  |  |
| Low (Tertile 1) | 206/860 | 1.00 (0.80 to 1.26) | 0.82 (0.65 to 1.05) | 0.84 (0.65 to 1.08) |
| Middle (Tertile 2) | 187/858 | 0.93 (0.74 to 1.18) | 0.85 (0.67 to 1.08) | 0.86 (0.67 to 1.10) |
| High (Tertile 3) | 193/859 | 1 (Ref.) | 1 (Ref.) | 1 (Ref.) |

OR, odds ratio; CI, confidence interval

^a^ The definitions of prediabetes, diabetes, and normoglycemia were based on the American Diabetes Association criteria.

^b^ Unadjusted.

^c^ Adjusted for age and sex.

^d^ Adjusted for age, sex, a body shape index, history of smoking and drinking, presence of hypertension, dyslipidemia, and heart disease, systolic blood pressure, diastolic blood pressure, triglycerides, total cholesterol, low-density lipoprotein-cholesterol, high-density lipoprotein-cholesterol, high-sensitivity C-reactive protein, and hemoglobin A1c at baseline.

^e^ Incomplete data mainly included age, body mass index, waist circumference, blood pressure, triglycerides, total cholesterol, high-density lipoprotein-cholesterol, and low-density lipoprotein-cholesterol.

# Supporting Information Figure S1. Subgroup analyses for the association of normalized grip strength with prediabetes regression and progression.

OR, odds ratio

Multinomial logistic regression analysis was conducted to obtain the ORs and 95% confidence intervals for the association of normalized grip strength in tertiles with progression to diabetes or regression to normoglycemia based on the American Diabetes Association criteria, with participants remained as prediabetes during follow-up serving as the base. All the analyses were controlled for age, sex, a body shape index, history of smoking and drinking, presence of hypertension, dyslipidemia, and heart disease, systolic blood pressure, diastolic blood pressure, triglycerides, total cholesterol, low-density lipoprotein-cholesterol, high-density lipoprotein-cholesterol, high-sensitivity C-reactive protein, and hemoglobin A1c at baseline.

# Supporting Information Figure S2. Subgroup analyses for the association of chair-rising time with prediabetes regression and progression.

OR, odds ratio

Multinomial logistic regression analysis was conducted to obtain the ORs and 95% confidence intervals for the association of chair-rising time in tertiles with progression to diabetes or regression to normoglycemia based on the American Diabetes Association criteria, with participants remained as prediabetes during follow-up serving as the base. All the analyses were controlled for age, sex, a body shape index, history of smoking and drinking, presence of hypertension, dyslipidemia, and heart disease, systolic blood pressure, diastolic blood pressure, triglycerides, total cholesterol, low-density lipoprotein-cholesterol, high-density lipoprotein-cholesterol, high-sensitivity C-reactive protein, and hemoglobin A1c at baseline.
